# First person – Rujuta Deshpande

**DOI:** 10.1242/dmm.049597

**Published:** 2022-05-09

## Abstract

First Person is a series of interviews with the first authors of a selection of papers published in Disease Models & Mechanisms, helping early-career researchers promote themselves alongside their papers. Rujuta Deshpande is first author on ‘ TOR signalling is required for host lipid metabolic remodelling and survival following enteric infection in *Drosophila*’, published in DMM. Rujuta conducted the research described in this article while a PhD student in Dr Savraj Grewal's lab at Clark H. Smith Brain Tumour Centre, Arnie Charbonneau Cancer Institute, and Department of Biochemistry and Molecular Biology, Cumming School of Medicine, University of Calgary, Calgary, Canada. She is now a Postdoctoral Associate in the lab of Dr Derek McKay at Calvin, Phoebe & Joan Snyder Institute for Chronic Diseases, Department of Physiology & Pharmacology, Cumming School of Medicine, University of Calgary, investigating the role of human interleukin (IL)4-activated macrophages in wound healing of the intestinal epithelium as a new treatment for inflammatory bowel diseases (IBD).

**Figure:**
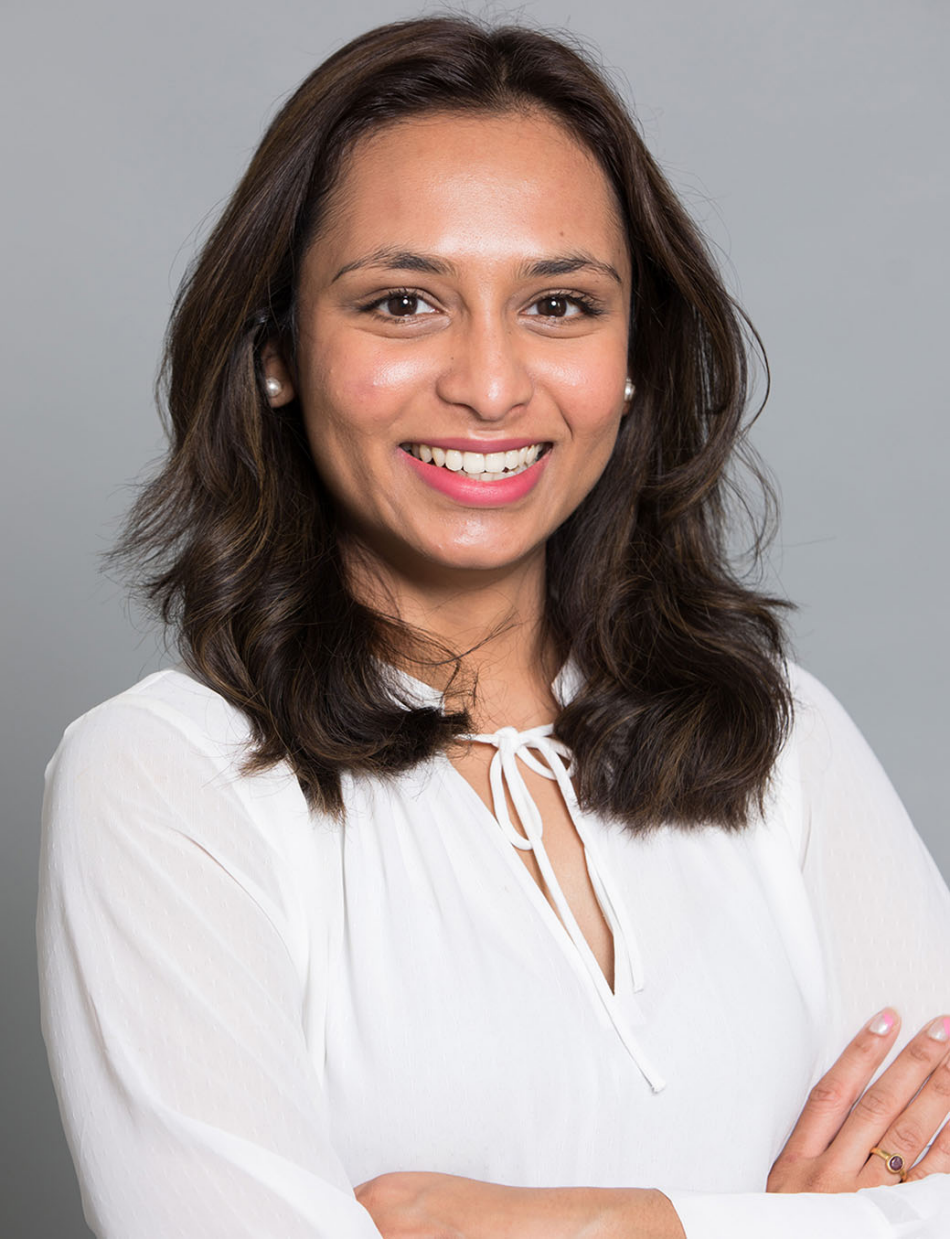
Rujuta Deshpande

**How would you explain the main findings of your paper to non-scientific family and friends?**

Animals in their natural ecology are often exposed to environmental stressors (e.g. starvation, extreme temperature, hypoxia, pathogens) that can affect their physiology, development and lifespan. An important question in biology is how animals sense these stresses and, in response, adapt their metabolism to maintain homeostasis and survival. The stress responses and metabolic adaptations are remarkably similar across different species such as humans and fruit flies. In response to oral pathogenic bacterial infection, the fruit fly responds by both local and systemic anti-bacterial immune responses to resist infection, and metabolic changes to promote infection tolerance. Here, we show that one way that the fruit fly mediates adaptive metabolic responses is via induction of target-of-rapamycin (TOR) kinase signalling. TOR is a well-established growth pathway and a regulator of metabolism. Our data support a model in which induction of TOR signalling in the fly intestine represents a host adaptive response to counteract infection and promote survival.

**Figure:**
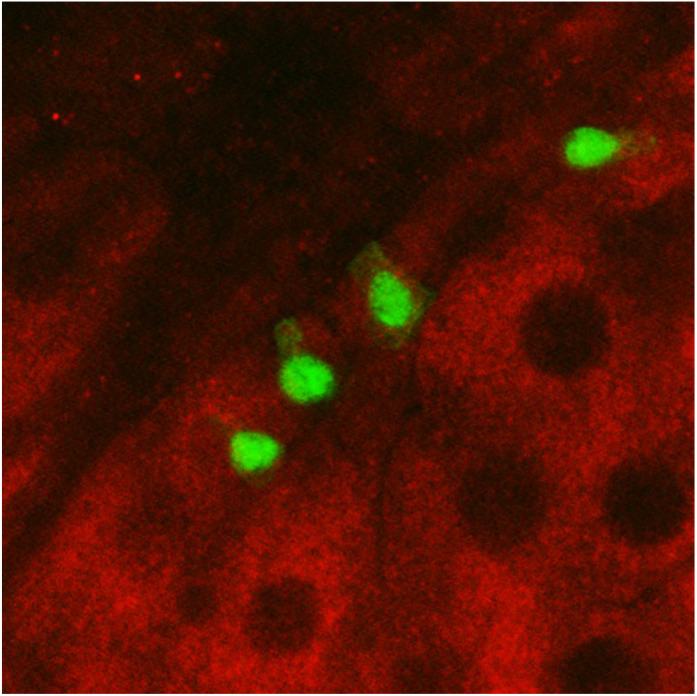
Immunofluorescence staining of the *Drosophila* intestine, showing that the TOR signalling pathway readout, ribosomal protein phospho S6 (red), typically suppressed upon stressed conditions, is shown to be upregulated in the stem cells (green nuclei) as well as the large epithelial cells upon bacterial infection stress.

**What are the potential implications of these results for your field of research?**

The innate immune system and the ability of the host to counteract the pathogenic bacteria (resistance mechanisms) upon infection has been studied extensively over the years. However, little is known about the tolerance adaptations that the host employs to survive the existing infection. Metabolic reprogramming is one such tolerance mechanism adapted by the host to promote survival. Our data support a model in which, upon infection, induction of TOR signalling helps promote infection tolerance by remodelling of the host lipid metabolism, independent of the immune signalling. Further detailed studies using fruit fly, and higher model systems, could potentially explain the inter-individual differences in their ability to respond to infections and aid host fitness. The outcomes of these studies may help to develop individualized treatments, taking into consideration both the resistance and tolerance abilities of the host.

**What are the main advantages and drawbacks of the model system you have used as it relates to the disease you are investigating?**

*Drosophila* is an inexpensive model organism with a short life cycle and has proven to be a powerful system to study host defence responses to infections. The conserved immune as well as metabolic pathways, although simplified compared to humans, are very well characterized. The availability of a variety of genetic and pharmacological tools to alter these pathways makes *Drosophila* a simple yet dynamic system for studying the responses to different stresses, particularly infection. Moreover, the ease of studying tissue-specific and systemic changes provides an adequate understanding of the inter-organ interactions developing in the host upon disease progression.

One of the major drawbacks of studying immune responses in *Drosophila* is the lack of an adaptive immune system. Although the simplicity of the system makes it easier to study the underlying mechanisms, it is not possible to study the later secondary adaptive responses to bacterial and other infections. These secondary responses, such as the memory response to certain pathogens and its systemic effects, are known to affect the outcome if the infection is encountered again.

**What has surprised you the most while conducting your research?**

TOR signalling is a well-established regulator of metabolism that has classically been shown to be activated by growth cues and suppressed by stress conditions. Interestingly, however, we found a rapid increase in TOR activity in the fly gut in response to bacterial infection stress. Our study shows a previously unappreciated differential role of TOR signalling upon different stress conditions, in particular bacterial infection stress. Furthermore, our study highlights the importance of using a simple model system which can potentially uncover mechanisms for understanding the inter-individual differences in response to infections.

**Describe what you think is the most significant challenge impacting your research at this time and how will this be addressed over the next 10 years?**

The most significant challenge working with *Drosophila* is the scalability of the research, or the bench-to-bedside aspect. Although, a majority of the signalling pathways studied are conserved across species, they are more complicated, with appreciably more interactions and genetic complications, in higher model systems. Studying the pathway interactions in *Drosophila* gives only fundamental insights of the biology and much work is needed for application-based medical research.

Despite the lack of direct bench-to-bedside scalability, some studies have now begun using *Drosophila* for development of disease mechanisms and personalized cancer therapeutics. For example, Dr Ross Cagan's laboratory at the Institute of Cancer Sciences, University of Glasgow, UK, is working towards a fly-to-bedside approach for treating cancer patients using *Drosophila* to identify personalized drug cocktails. Such an approach can hopefully lead the way for future fly-to-bedside therapeutics for treatments of immunological and inflammatory diseases as well.

**What changes do you think could improve the professional lives of early-career scientists?**

I think increased opportunities to present their work on a larger platform with people working in similar fields would help early-career researchers to be more critical of their work. Apart from improved scientific communication opportunities, increased funding is essential for early-career scientists to be able to maintain a good professional-personal life balance and continue to pursue answers to intriguing and impactful scientific questions.

**What's next for you?**

I have always been intrigued by the dynamic nature of the intestine, where it acts an important barrier, a host of several immune cells and a diverse microbiome, all working synchronously in maintaining homeostasis and host fitness. I have continued to pursue my interest in studying this fascinating organ in a different model system for my postdoctoral studies. I am currently working on an exciting project in the McKay laboratory at the University of Calgary, which focusses on the advanced studies the role of human IL4-activated macrophages in wound healing of the intestinal epithelium. It is part of a larger project where we intend to treat IBD by treating the patients with an autologous cell transfer of their own IL4-activated and cryopreserved macrophages upon disease relapse.
